# Understanding adipocyte heterogeneity across species, depot, and disease

**DOI:** 10.1042/BST20250327

**Published:** 2026-07-24

**Authors:** Elizabeth S. Kitto, Shiri Kult Perry, Margo P. Emont

**Affiliations:** Department of Medicine, Section of Endocrinology, Diabetes, and Metabolism, University of Chicago

**Keywords:** adipocytes, brown adipose tissue, obesity, single nuclear rna sequencing, type 2 diabetes, white adipose tissue

## Abstract

Adipose tissue is a key regulator of metabolic homeostasis; however, its dysfunction can lead to complications, including the development of metabolic disease. While mature adipocytes play an important role in this relationship, their size and buoyancy have made characterizing them difficult at the single cell level. Development of single-nuclear RNA sequencing and spatial transcriptomics have allowed for the study of mature adipocytes at unprecedented resolution and have enabled the identification of previously unappreciated subpopulations of adipocytes, some of which are associated with specific adipose depots or metabolic conditions. Here, we review the recent publications in the field and attempt to synthesize the populations identified by individual studies into classes based on their predicted functionality. We also discuss critical gaps in our current understanding of adipocyte subpopulations, including the current dearth of functional characterization of these populations.

## Introduction

Adipose tissue is a dynamic organ essential for energy storage, temperature regulation, hormone secretion, and mechanical protection of internal organs. It plays a central role in maintaining metabolic homeostasis, and its dysfunction contributes to chronic metabolic diseases such as obesity and type 2 diabetes [1]. Adipose tissue is distributed throughout the body and broadly classified into subcutaneous and visceral depots, each composed of heterogeneous cell populations [2]. In humans, these include adipocytes, blood and lymphatic vessels, immune cells, and adipose stromal and progenitor cells (ASPCs), which are comprised of preadipocytes (PreA, ICAM1+), adipose stem and progenitor cells (ASPCs, DPP4+), and Aregs (CD142+) [3,4]. While it was once thought that mature adipocytes themselves were relatively uniform, recent studies have begun to define adipocyte subpopulations in greater detail, revealing transcriptionally and functionally distinct populations. Despite progress, the diversity and abundance of adipocyte subpopulations across species, health states, and environmental conditions requires further investigation.

Adipose tissue dysfunction drives the development of metabolic disease, increasing morbidity and mortality and elevating the risk of comorbid conditions such as coronary heart disease, stroke, and MASLD (metabolic dysfunction-associated steatotic liver disease) [5]. These health consequences highlight the need to understand how adipose tissue biology and mature adipocytes specifically shape systemic metabolism. Do specific adipocyte subtypes promote health or disease? Are variations in adipocyte populations driven primarily by environmental exposures such as diet and temperature, or by genetic factors? And can these populations be therapeutically modified to improve metabolic outcomes? In the present review, we highlight recent studies characterizing adipocyte heterogeneity through classical approaches and sequencing-based techniques.

## Methods of studying heterogeneity in adipocytes

The development and widespread use of single-cell and single-nucleus RNA sequencing (sc and snRNA-seq, respectively) has transformed the study of adipose tissue by enabling unbiased analysis of transcriptional differences between individual cells [6]. scRNA-seq studies of the stromal vascular fraction have revealed diverse populations of preadipocytes, mesenchymal progenitors, immune cells, endothelial cells, and other stromal components that remodel across metabolic states [7–11]. However, the large size and high lipid content of adipocytes results in a buoyant, fragile cell that is challenging to sequence with microfluidic devices or flow sorting methods used for scRNA sequencing. By contrast, snRNA-seq circumvents these issues by profiling isolated nuclei, typically capturing adipocytes at proportions much closer to their true abundance within adipose tissue [6,12]. As a result, the field is increasingly relying on snRNA-seq, often in combination with classical techniques such as imaging, to generate a more complete picture of adipocyte heterogeneity.

**Figure 1:**
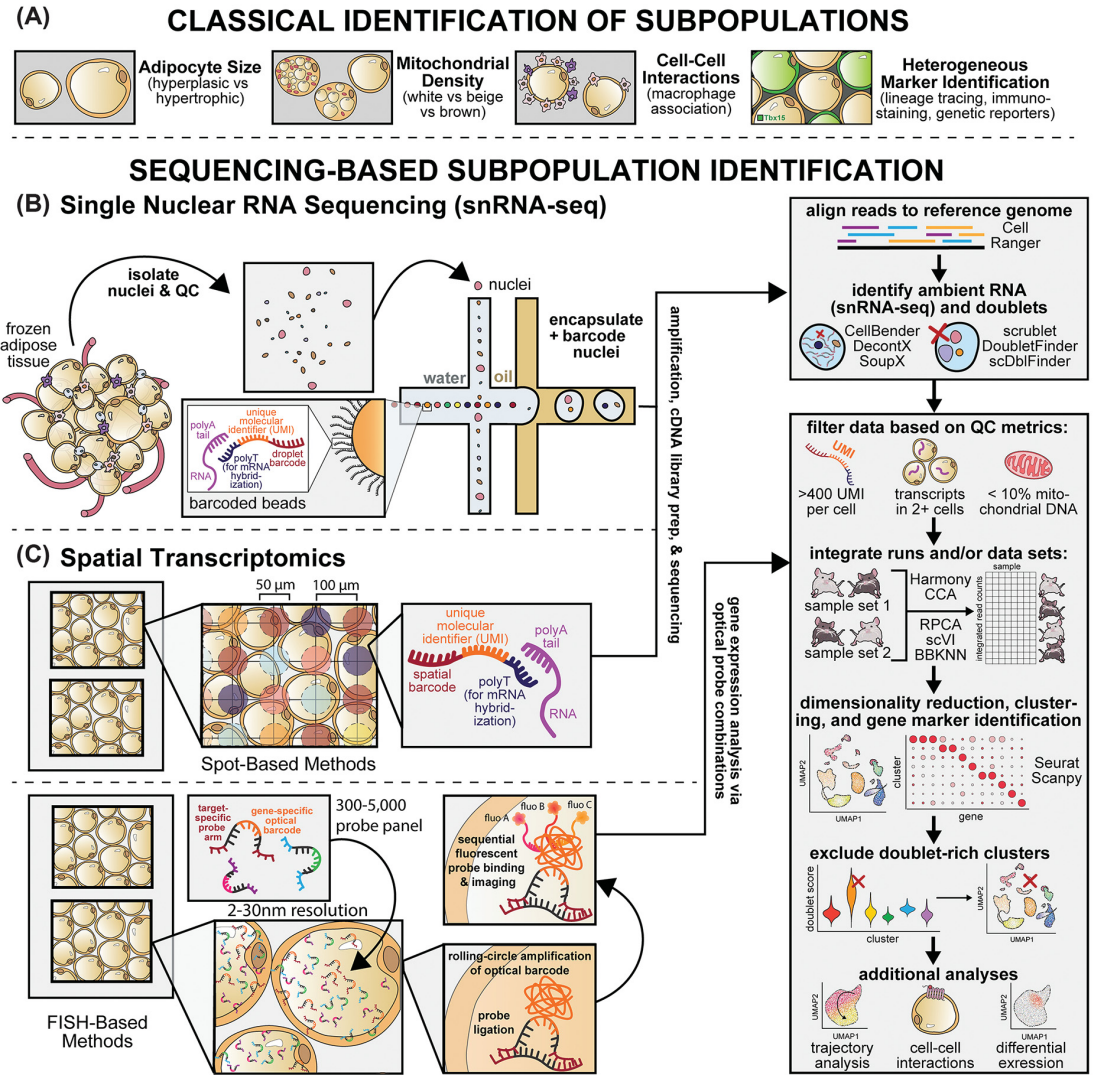
Methods of studying adipocyte heterogeneity (**A**) Classical methods of studying adipocyte heterogeneity include grouping cells by morphological characteristics like size, mitochondrial density, proximity to other cell types, or transgenic markers of differentially expressed genes. (**B,C**) Three common sequencing approaches to studying adipocyte heterogeneity include snRNA-seq (**B**), or spatial transcriptomics like spot-based and Fluorescence *In Situ* Hybridization (FISH)-based methods (**C**).

However, even before these technologies became available, researchers studied adipocyte heterogeneity through differences in morphology, anatomical depot, cell-cell associations, and expression of selected marker genes or proteins [1,2,13] (Figure 1A). These approaches remain highly informative and continue to complement modern sequencing-based methods, particularly for functional validation and mechanistic follow-up. Below, we first summarize classical approaches to studying adipocyte heterogeneity, and then discuss newer sequencing-driven methods.

## Classical methods

### Heterogeneity by cell morphology and depot type

One of the earliest recognized examples of adipocyte heterogeneity is the distinction between white and brown adipocytes. Brown adipocytes are multilocular, containing numerous small lipid droplets and a high density of iron-rich mitochondria that confer their darker appearance and thermogenic capacity. White adipocytes, in contrast, are unilocular, with a single large lipid droplet and fewer mitochondria. In both humans and mice, classical brown adipose tissue (BAT) and white adipose tissue (WAT) occupy distinct anatomical depots. The location of BAT in humans also varies across the lifespan: newborns have BAT in the intrascapular region, which involutes during childhood [14], while thermogenic fat has been found in the supraclavicular region of adults [15–18]. Historically, these morphological and anatomical differences across depots enabled relatively straightforward dissection and comparative analysis [1,2]. In fact, BAT appears so morphologically distinct from WAT that it was initially identified in marmots in the 16^th^ century but was described as a completely separate organ, or ‘hibernation gland’ [19]. In the mid-20th century, isolation of BAT based on its morphology and localization led to the discovery that BAT exhibits dramatically elevated tissue respiration and thermogenesis relative to WAT [20]. Subsequent biochemical and physiological studies identified uncoupling protein 1 (UCP1) as a key mediator of non-shivering thermogenesis in brown adipocytes, linking mitochondrial density and morphology to function [21].

Within WAT, heterogeneity has also been defined by anatomical depot, most notably between subcutaneous adipose tissue (SAT) and visceral adipose tissue (VAT). SAT is located beneath the skin, whereas VAT resides within the abdominal cavity, surrounding organs such as the intestines and liver [12]. Comparative studies have revealed depot-specific differences in adipocyte gene expression [22–25], lipolytic and lipogenic capacity [26,27], and responsiveness to hormonal and inflammatory cues [28]. Importantly, VAT expansion is more strongly associated with insulin resistance, dyslipidemia, and cardiometabolic disease than SAT expansion, underscoring the functional importance of depot-specific heterogeneity in adipocytes [2,29].

### Heterogeneity in adipocyte function

Beyond overt differences in morphology and depot, adipocyte heterogeneity also exists within morphologically similar cells in the same depot. A classic example is the identification of ‘beige’ adipocytes, a subset of activatable thermogenic adipocytes in white fat depots. After the mechanism of UCP1-mediated thermogenesis was established in BAT, it was discovered that a subset of adipocytes from white adipose depots could upregulate UCP1 and other BAT selective genes in response to cold exposure or adrenergic stimulation, despite having minimal basal UCP1 expression [30]. Transgenic mouse lines or cell models with reporters for *Ucp1* expression have been used in combination with other specific markers (*Chrna2, Cd137, Tmem26*) to identify subpopulations of beige adipocytes while immunostaining has validated these heterogeneous expression patterns at the protein level [30,31]. The identification of beige adipocytes illustrates how examining heterogeneity in small sets of marker genes or proteins can uncover functionally distinct adipocyte subtypes.

In addition, live-cell imaging approaches, including calcium imaging or staining for activation of specific signaling pathways, have been used to distinguish ‘responder’ and ‘non-responder’ adipocytes to specific stimuli (e.g. acetylcholine receptor [31] or insulin signaling [32,33]), further highlighting functional heterogeneity within morphologically similar cells. Many of these approaches based on adipocyte function remain valuable today, particularly for validating subpopulations identified by sequencing.

### Heterogeneity in adipocyte size

Adipocyte size provides another example of morphological and functional heterogeneity, with large and small adipocytes within the same depot exhibiting distinct metabolic properties. Measuring adipocyte size using quantitative imaging methods has revealed that hypertrophic adipocytes (fewer, larger cells) are strongly associated with insulin resistance, obesity, and inflammation [34–36]. By contrast, a hyperplasic morphology (more numerous but smaller adipocytes) is generally linked to a more favorable metabolic profile, even at similar levels of total fat mass [36,37]. Separation of large versus small adipocytes based on differences in buoyancy further found that even under obesogenic conditions there is a heterogeneity of adipocyte size and associated distinct transcriptomic and lipidomic profiles in large versus small adipocytes [38].

### Heterogeneity in cell-cell interactions

Adipocytes also differ in their interactions with other cell types within adipose tissue. One striking example is the formation of ‘crown-like structures’ [39,40], in which macrophages encircle dead or dying adipocytes. Adipocytes that are closely associated with these inflammatory macrophages exhibit distinct transcriptional and secretory profiles, including altered adipokine production and increased stress signaling [40–42]. While adipocytes associated with these crown-like structures exhibit greater inflammatory gene expression, the directionality of this interaction remains unclear. More broadly, proximity to vascular cells [43,44], nerve fibers [45–47], or specific immune cell subsets [48–50] can influence adipocyte phenotype and function. Classically, adipocyte heterogeneity in cell-cell interactions has been identified via immunostaining-based imaging methods, but modern spatial omics approaches have the potential to directly connect cell type interactions with transcriptomic data [11,51].

## Sequencing-based methods

### Single-nucleus RNA sequencing

scRNA-seq rapidly became a foundational method for characterizing cellular heterogeneity across many tissue types. In adipose tissue, scRNA-seq studies have uncovered diverse stromal cell populations [4,52,53] and dynamic changes in cellular composition across lean, obese, weight-loss, and aged states [7,54,55]. However, the large, fragile, and buoyant nature of mature adipocytes leads to inconsistent recovery and biased sampling of adipocytes in whole-tissue datasets. snRNA-seq has therefore emerged as the primary transcriptomic approach to study heterogeneity among mature adipocytes, because nuclei can be robustly isolated from both fresh and frozen adipose tissue, regardless of cell size or fragility (Figure 1B).

Common downstream analyses of snRNA-seq include differential gene expression between clusters or conditions, pseudotime or trajectory analyses to infer developmental or remodeling pathways, and ligand-receptor interaction analyses to predict crosstalk between adipocytes and other cell types [6,12]. Despite its advantages, snRNA-seq has important limitations. Because it focuses on nuclear RNA, cytoplasmic and mitochondrial transcripts are not captured. snRNA-seq also lacks spatial information, and nuclear transcriptomes can differ from whole-cell or spatial datasets, making integration across different approaches challenging [11]. Finally, appropriate quality control measures are vital to correct subpopulation analysis, including ensuring a sufficient cell count for cell types of interest, removing doublets (when reads from two cells are combined, such as when two cells are in the same droplet) and correcting for ambient RNA (free RNA in the cell or nuclear preparation, particularly a problem in snRNA-seq when cytoplasmic RNA is released during nuclear isolation).

### Spatial transcriptomics

Spatial transcriptomics platforms provide gene expression measurements while preserving tissue architecture, making them well-suited to studying how adipocyte heterogeneity relates to depot structure, cell–cell interactions, and morphological features such as lipid droplet size. In adipose tissue, most published studies to date have employed spot-based methods such as the 10× Genomics Visium platform [56–58], which captures whole-transcriptome expression within barcoded spots with a ∼50 μm diameter. *In situ* hybridization-based platforms such as Xenium have also been applied to adipose tissue to provide more precise resolution (∼2–30 nm) for a targeted gene panel [51,59] (Figure 1C).

Spatial methods offer key advantages for studying adipocyte heterogeneity. Both types of spatial transcriptomics have contributed to direct linkage of adipocyte gene expression patterns to histological features, including adipocyte size [56,59], proximity to other cell types [56,60], and tissue architecture [58]. However, they also present challenges. Spot-based platforms have limited spatial resolution, and each spot may capture RNA from multiple adipocytes and non-adipocyte cell types, complicating deconvolution and interpretation. Read depth per spot can be relatively low compared with droplet-based snRNA-seq, limiting detection of lowly expressed genes. Moreover, integrating spatial and droplet-based datasets requires careful computational alignment, and discrepancies between nuclear and whole-tissue or *in situ* transcriptomes can complicate joint interpretation.

Together, traditional morphology-based approaches, single-gene and protein analyses, snRNA-seq, and spatial transcriptomics provide complementary perspectives on adipocyte heterogeneity. Leveraging multiple methods in combination is likely to be essential in determining how diverse adipocyte subpopulations arise, interact with their microenvironment, and contribute to metabolic health and disease.

## Unique adipocyte populations in mouse and human adipose tissue

An expanding body of work using snRNA-seq and spatial transcriptomics is redefining how we view adipocyte heterogeneity. Most studies focus on mouse and human adipose tissue, but analogous adipocyte subpopulations have also been described in other species, including cows [61], pigs [62] and chickens [63]. Subpopulations are primarily identified by clustering based on gene-expression, with validation via qPCR, immunostaining, and functional assays of adipocyte health. Collectively, these studies reveal distinct adipocyte populations that vary by anatomical depot, sex, metabolic health, and environmental conditions. Some clusters are linked to specific functions such as adaptive thermogenesis or lipid metabolism, whereas the functional roles of many others remain unclear. Consequently, comparing adipocyte populations across datasets and methodologies remains a major challenge [12]. One integrative effort by Massier et al. harmonized all cell types within adipose tissue except for adipocytes [11], highlighting the technical and biological challenges of cross-study comparison in mature adipocytes.

The question of cell state versus cell type further complicates this picture. For example, cold exposure [57,64–68] and obesity [10,59] alter the proportions of several adipocyte clusters, indicating that these populations are dynamic. However, it remains unresolved whether clusters represent transient transcriptional states that individual cells cycle through under specific conditions, or whether they constitute distinct cell types arising from different precursors. Both scenarios may also be true: some populations may derive from unique lineages while still being modified by environmental factors. Additionally, comparing adipocyte subpopulations across species is challenging due to differences in gene expression, gene homology, physiological function, depot anatomy and experimental characteristics [10,69].

In the present review, we summarize adipocyte subpopulations reported across 11 studies of human tissue and 18 studies of mouse tissue. Because marker genes for adipocyte subpopulations are inconsistent across studies and methodologies, we instead organized these clusters by their known or inferred function. Putative functional roles were assigned to adipocyte subpopulations using the following ranked criteria: (1) functional roles explicitly defined in the text or main figures; (2) top functional pathways from cluster-specific KEGG or GO analyses; and (3) Subpopulation-specific genes associated with a functional category. If a subpopulation met these criteria for three or more functional categories, the top two categories with the strongest evidence were included in the figure and tables.

In human WAT, subpopulations fall into six phenotypic categories: (1) lipid metabolism, (2) adaptive thermogenesis, (3) inflammation, (4) hypoxia/fibrosis, (5) anabolic/cell growth, and (6) signaling. Mouse studies of WAT further divide the lipid metabolism category into (1a) lipolytic, (1b) lipogenic, and (1c) lipolytic and lipogenic populations. The greater availability of mouse studies also allows the adaptive thermogenesis category to be classified into (2a) UCP1 adaptive thermogenic and (2b) futile cycling adaptive thermogenic populations.

In murine BAT, four subpopulations have been described and are associated with (1) high UCP1, (2) intermediate UCP1, (3) futile cycling, and (4) thermoregulatory phenotypes. Subpopulations of murine beige adipocytes have also been reported, falling into the categories of UCP1 dependent and UCP1 independent thermogenic adipocytes. From the limited data on human brown adipocyte heterogeneity, it remains unclear whether there are multiple subpopulations of human thermogenic adipocytes.

In both human and mouse datasets, some subpopulations are enriched in specific adipose tissue depots or under certain environmental conditions like temperature or diet. Details about common marker genes used to identify subpopulations within these categories and associations of each population with depot and metabolic health can be found in Supplementary Tables S1–S3. Notably, some reported subpopulations span multiple functional categories, others are unique to a single study, and still others have undefined phenotypic characteristics. Future work must clarify these open questions, refine cross-dataset and cross-species comparisons, and functionally validate the roles of clusters inferred by transcriptomic data.

## Distinct populations of white adipocytes

**Figure 2:**
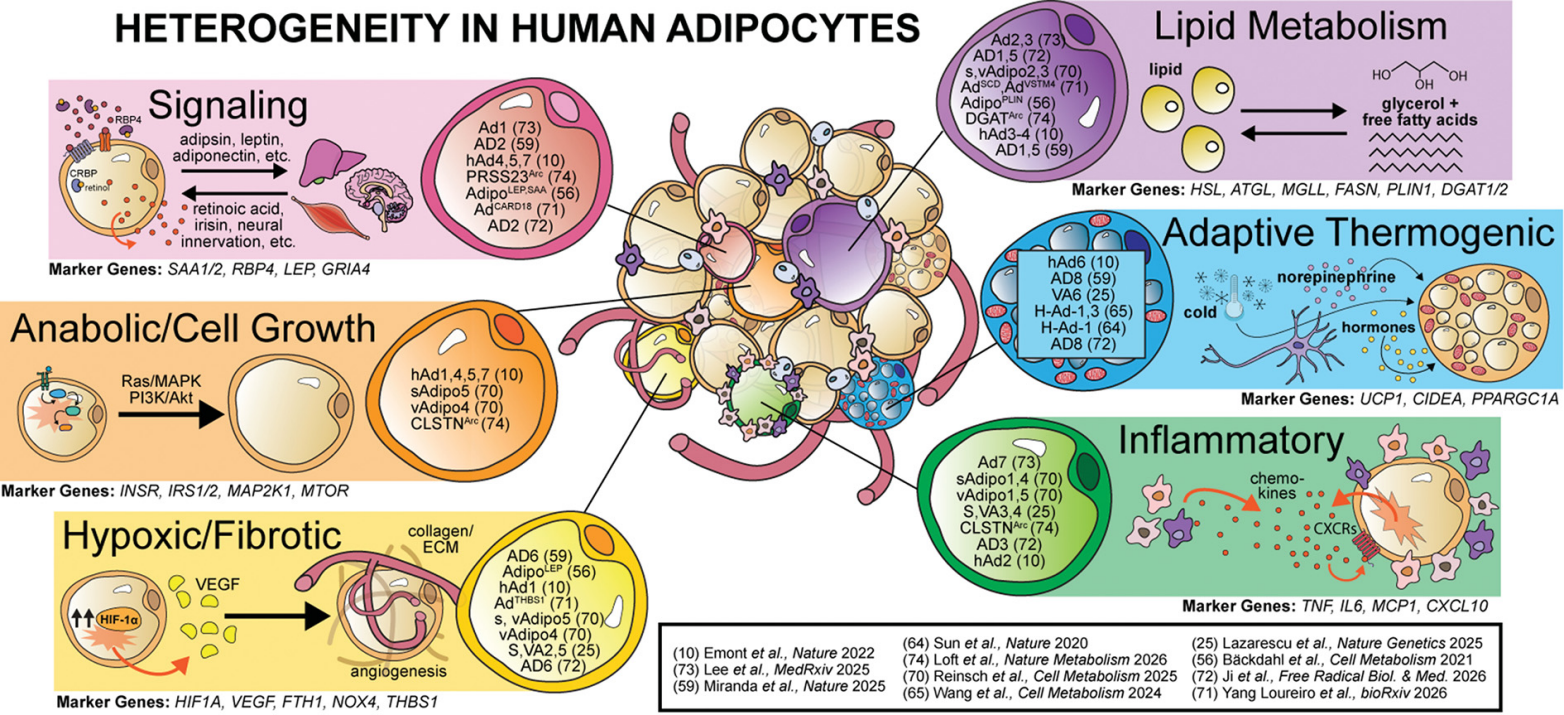
Human adipocyte subpopulations identified by single-nuclear and spatial transcriptomics across eleven studies of adipose tissue Across 11 studies of human adipose tissue [10,25,56,59,64,65,70–74] (11 with subcutaneous samples, 3 with visceral samples, and 2 with deep neck samples), single-nuclear and spatial transcriptomic approaches have identified transcriptionally distinct adipocyte populations associated with lipid metabolism, adaptive thermogenesis, signaling, anabolic or growth-related pathways, and cellular stress responses.

**Figure 3:**
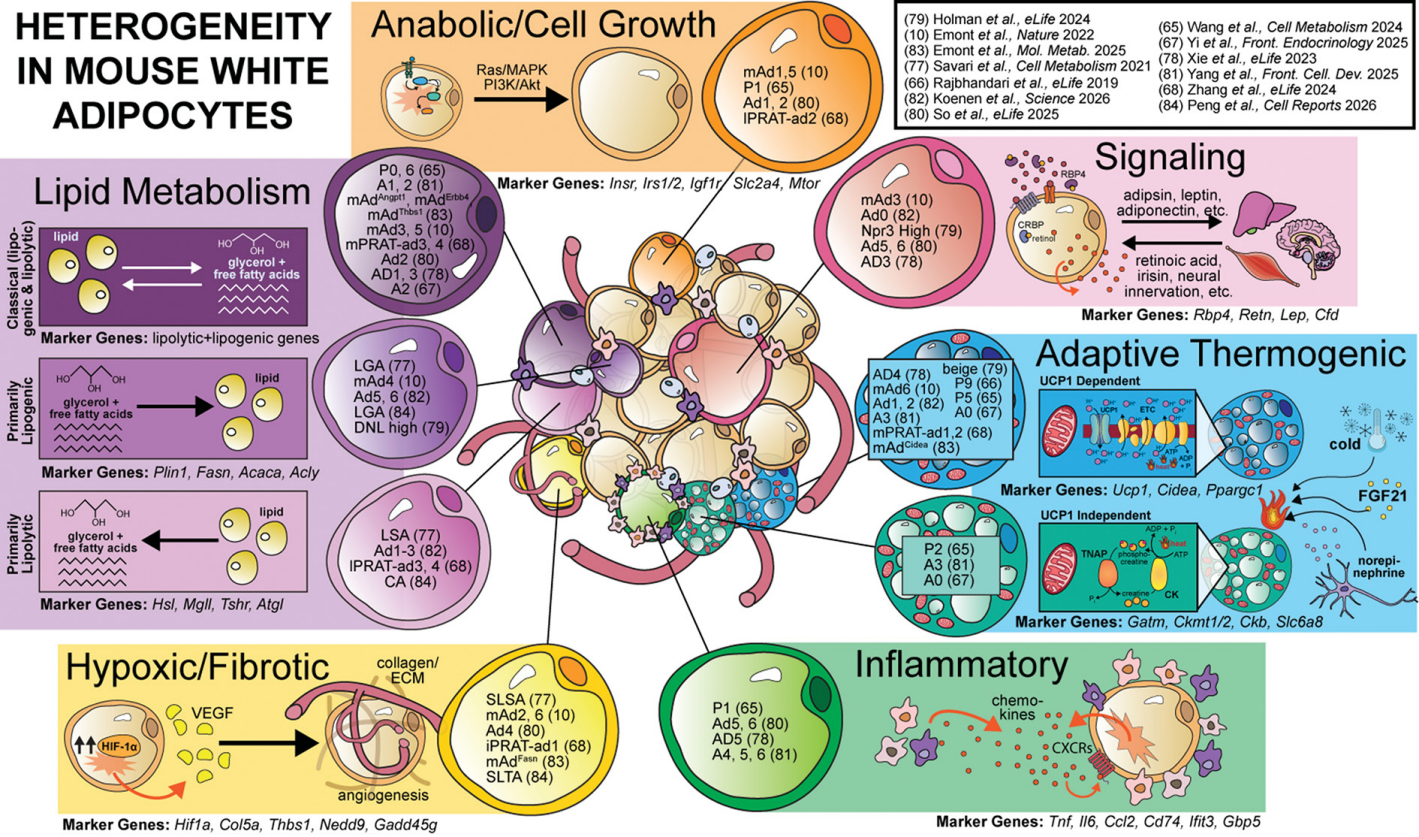
Mouse adipocyte subpopulations identified by single-nuclear and spatial transcriptomics across 13 studies of white adipose tissue Across 13 mouse studies [10,65–68,77–84], WAT has been shown to contain multiple transcriptionally distinct adipocyte populations spanning lipid metabolism, adaptive thermogenesis, signaling, anabolic or growth-related programs, and stress-associated states, including inflammatory and hypoxic/fibrotic clusters.

**Figure 4:**
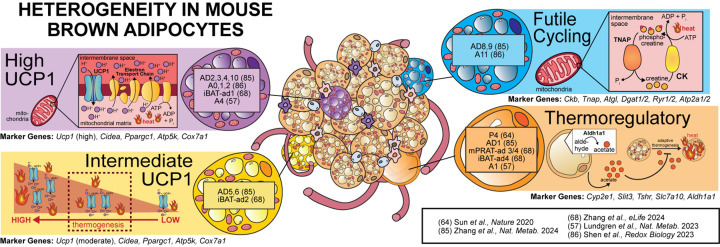
Mouse adipocyte subpopulations identified by single-nuclear and spatial transcriptomics across five studies of mouse brown adipose tissue Five studies of mouse BAT [57,64,68,85,86] have defined several adipocyte subpopulations, including high-UCP1 and intermediate-UCP1 clusters, UCP1-independent futile-cycling populations, and thermoregulatory cells with limited thermogenic gene expression.

In this functional categorization of human adipocytes (Figure 2), we include populations from 11 human snRNA-seq and spatial transcriptomics studies of WAT that both (1) report adipocyte subpopulations, and (2) contain >2 patient samples [10,25,56,59,64,65,70–74]. Additional studies of human WAT that do not meet this criterion are included in Supplementary Table S4 [11,75,76]. These studies include diverse cohorts differing in sex, depot (subcutaneous versus visceral), BMI, and metabolic health (lean, obese, type 2 diabetes, post-bariatric surgery). When categorizing mouse adipocyte heterogeneity, we have included adipocyte populations from 18 snRNA-seq and spatial transcriptomics studies of white [10,65–68,77–84] (13 studies) and brown [57,64,68,85,86] (5 studies) adipose tissue. All murine studies that analyzed adipocyte subclusters were included (Figures 3, 4, Supplementary Tables S5,S6). Murine studies cover many of the same variables as human studies, such as sex, diet/metabolic status, and age, as well as additional depots, genetic modifications, and cold exposure conditions. Here, we focus on conditions present in wild type mice, although the abundance of some may be influenced by genetic manipulation.

### Lipid metabolism and cell growth subpopulations

Most single-cell and single-nucleus adipose datasets identify at least one ‘canonical’ or ‘basal’ adipocyte population characterized by high expression of genes central to adipocyte functions such as lipolysis (e.g., *LIPE, MBLP, ATGL/PNPLA2*) and lipogenesis (e.g., *FASN, ACACA, DGAT1, SCD1*). These canonical adipocytes typically constitute a large fraction of total adipocytes. Several reports in human adipose tissue additionally describe subpopulations that express lipolytic genes but are defined predominantly by high expression of lipogenic genes alone (Ad5 [59], s,vAdipo3 [70]). This distinction between primarily lipogenic and primarily lipolytic subpopulations is more often observed in the mouse literature, although many publications still report canonical populations defined by general lipid metabolism. Primarily lipolytic or lipogenic clusters may reflect cells actively engaged in lipid storage or nutrient mobilization at the time of sequencing. In some studies they may also be primarily defined by the absence of the less-traditional gene programs characteristic of other adipocyte subclusters.

Many studies also identify adipocyte subpopulations enriched for cell-growth pathways, including Ras/MAPK and PI3K/Akt signaling components. Interestingly, these growth-associated clusters do not necessarily overlap with lipid metabolism populations (Ad1 [80], P1 [65], sAdipo5 [70], vAdipo4 [70], hAd5 [10]). This suggests that while developing adipocytes do express lipid metabolism genes, they are not yet major contributors to the key lipid handling processes seen in the mature adipocytes.

### Signaling subpopulations

Adipose tissue functions as a key endocrine organ, communicating with liver, muscle, brain, and other tissues to regulate whole-body metabolism. snRNA-seq and spatial studies reveal that not all adipocytes contribute equally to this signaling network. Some subpopulations are defined by high expression of adipokines such as *CFD* (adipsin), *ADIPOQ* (adiponectin), and *LEP* (leptin), suggesting specialized roles in systemic energy sensing and metabolic regulation. Other clusters show elevated expression of receptors for peripheral signals, including glutamate receptors (*GRIA4*), retinoic acid-binding proteins (*RBP4*, *CRBPS*), and the GIP receptor (*GIPR*). These receptor-enriched adipocytes may be particularly sensitive to circulating cues and could drive state-specific changes in gene expression.

### Stressed subpopulations (hypoxic/fibrotic, and inflammatory)

Not all adipocyte subpopulations reflect healthy function; many are defined by markers of cellular stress that arise during overnutrition and metabolic disease. Adipose tissue expansion often outpaces vascular growth, thus a hypoxic response as well as angiogenic signaling genes can be seen in some adipocytes, as well as fibrosis and ECM deposition genes to facilitate tissue growth. Some but not all adipocyte subpopulations are characterized by high expression of these pathways in adipose tissue from both metabolically healthy and unhealthy individuals, indicating that some hypoxia is seen even under healthy conditions. Further spatial work can characterize the tissue architecture in different metabolic states to potentially explain the relationship of these populations to the vasculature.

A second population of ‘stressed’ adipocytes are characterized by increased inflammatory signaling, including elevated expression of pro-inflammatory cytokines (e.g., *TNF*, *IL6*, *CCL2*) and receptors for inflammatory signals. This inflammatory cluster does not completely overlap with hypoxic/fibrotic clusters, suggesting these two subpopulations could represent a progression of the adipocyte stress response, or two distinct adaptations to a stressful environment. Interestingly, while overall adipose tissue inflammation is strongly associated with obesity and metabolic disease [1,87], these inflammatory populations are seen across the spectrum of BMI and metabolic health examined in the current studies.

### Adaptive thermogenic subpopulations

Thermogenic adipocytes have previously been described in both human and mouse white fat depots [15–18,30]. These cells do appear as distinct subclusters in snRNA-seq data of WAT, characterized by expression of classical thermogenic markers such as *Ucp1*, *Cidea*, *Dio2*, and *Ppargc1a*. In mouse WAT, distinct subpopulations of beige adipocytes have been identified based on their primary mechanism of thermogenesis (*Ucp1* dependent or independent, more details in section on Mouse Brown Adipose Tissue). It remains an open question what the relationship is between these subpopulations and if further distinctions might be found based on preferential use of distinct UCP1-independant mechanisms (i.e. calcium cycling versus creatine cycling). The identification of distinct cellular lineages for murine thermogenic adipocytes (*Trpv1+* and *Pdgfra+* APCs) [88] also supports the idea that there are multiple subpopulations of thermogenic adipocytes.

The extent of heterogeneity among human thermogenic adipocytes remains unclear, particularly regarding whether, like murine adipocytes, they can be subdivided based on thermogenic programs. While human thermogenic adipocytes express genes related to both UCP1-dependent and -independent thermogenesis, current studies lack the statistical power to determine if these occur in distinct cell types. Additionally, no snRNA-seq study has examined human adipocyte subpopulations before and after controlled cold exposure. However, Wang et al. demonstrated strong similarity between gene-expression signatures of cold-induced brown adipocytes in mice and a human adipocyte subpopulation [65], suggesting that human thermogenic populations may also expand or shift in response to environmental cues. Interestingly, while most studies of human thermogenic fat have focused on deep neck and SAT, some studies identified thermogenic adipocytes in visceral samples [10], suggesting that studies that aim to identify and quantify these cells in humans should consider all depots.

## Distinct populations of mouse brown adipocytes

### UCP1 thermogenic subpopulations (high UCP1 and intermediate UCP1)

UCP1-mediated thermogenesis is the canonical mechanism of brown adipocyte function. UCP1, located in the inner mitochondrial membrane, dissipates the proton gradient generated by the electron transport chain, converting respiratory energy into heat rather than ATP. Within classical brown adipose depots, not all adipocytes express *Ucp1*, and *Ucp1*-expressing cells often subcluster into multiple populations in snRNA-seq studies. These are frequently categorized by relative thermogenic gene expression as ‘high UCP1’ and ‘intermediate UCP1’ thermogenic cells. High UCP1 clusters typically show strong expression of *Ucp1*, *Cidea*, *Dio2*, *Ppargc1a*, and fatty-acid oxidation genes, consistent with robust thermogenic capacity. Intermediate UCP1 clusters express lower but detectable levels of these markers and may represent less active thermogenic cells, transitional states, or distinct subtypes with partial thermogenic capacity.

### Futile cycling and thermoregulatory subpopulations

Not all cells present within mouse brown fat are enriched for classical thermogenic genes. As in murine beige adipocytes, studies have found a futile cycling subpopulation of brown adipocytes that is *Ucp1*-low but enriched for other thermogenic pathways such as futile creatine and calcium cycling. Additionally, in the first snRNA-seq study of murine intrascapular BAT, Sun et al. identified an adipocyte subpopulation (P4) that decreases in proportion following cold exposure [64]. This cluster generates acetate as a signal to inhibit thermogenesis in adjacent adipocytes and shows little to no thermogenic gene expression. Subsequent studies have found similar subpopulations in mouse iBAT and medial perirenal adipose tissue [68]. These thermoregulatory adipocytes may function to temper excessive thermogenesis and maintain energy balance under thermoneutral conditions, representing a previously unrecognized regulatory layer within brown fat.

## Discussion

snRNA-seq and spatial transcriptomics have offered unprecedented resolution into adipocyte heterogeneity, finding that adipose tissue contains multiple, functionally diverse adipocyte subpopulations in both white and brown depots, across species and metabolic states. Some clusters are tightly linked to classic adipocyte functions (lipid storage, thermogenesis), while others highlight stress responses, signaling specialization, or regulatory roles. However, our understanding of adipocyte diversity is still limited by the availability of data, particularly in humans. Most human studies have been performed on abdominal subcutaneous fat, omental fat, or cervical fat, but snRNA-seq datasets have begun to be generated on other depots such as perivascular [89] and epiploic [90] adipose tissue. Subclustering of adipocytes from these and other depots will provide further insight into the similarities of adipocyte subclusters across depot and potentially identify new adipocyte subclusters.

Additionally, the conditions studied in humans have been limited to weight and diabetes status. The present work has provided correlative information on which adipocyte subpopulations increase or decrease with changes in environmental factors or metabolic health parameters. For example, combining GWAS associations for metabolic disease with snRNA-seq data suggested a specific adipocyte subpopulation (hAd7) may be associated with type 2 diabetes [10]. Other studies have identified adipocyte populations that decrease following weight loss (Ad1 [73], Ad^CARD18^ [71]) and some adipocyte subclusters also correlate with disease outcomes, such as metabolically unhealthy obesity (sAdipo5 [70]) or type 2 diabetes (AD3 [72], H-Ad-1 [65]). Using mouse models has also allowed for studies examining changes in adipocyte subpopulations following interventions such as cold exposure or a high fat diet (A1, A4 [57], and P0, P6 [65] in response to cold; Ad5, Ad6 [80] and mAd1,3,4, and 5 [10] in response to HFD).

Despite these correlative findings, there is limited evidence for direct causality, which remains a primary goal for future research. Validating the causal role of a subpopulation in disease outcomes will require functional assays to be performed on specific adipocyte subpopulations to more directly point to a disease association. Rodent studies have made progress by deleting subpopulation-specific marker genes to directly demonstrate effects on metabolic health [64,85]. However, more work will need to be done to assess the function of already identified adipocyte subpopulations and establish any causal effects of these subpopulations on disease states.

Other key challenges also remain, such as defining whether clusters represent stable cell types or transient states, establishing cross-species homology, integrating snRNA-seq with spatial and functional data, and experimentally validating the physiological roles of less-characterized subpopulations. The fact that cold exposure, obesity, aging, and depot-specific cues can shift the proportion of particular clusters argues that adipocyte identity is dynamic. At the same time, some populations may arise from distinct developmental lineages and then be further modified by environmental inputs, making strict ‘cell type versus cell state’ categories oversimplified. A third major challenge is in comparing human and mouse adipocytes. Mouse data is critical to examining how manipulating environmental and genetic factors affects adipose tissue heterogeneity, but while similar categories of adipocytes can be found in both mouse and human datasets, efforts to harmonize subclusters based on gene expression have not been successful [10]. This makes it difficult to perform experiments examining the development and function of human adipocyte subclusters.

Future progress in understanding adipocyte heterogeneity will require standardized dataset generation and quality control, and broad open access to raw and processed data and metadata. Equally important will be experiments that move beyond transcriptionally-based clustering to testing function directly, including integrative analyses that combine transcriptomics with histology, spatial context, and functional assays. Because the available spatial transcriptomic data on adipose tissue is limited, future studies can expand on our current spatial knowledge and combine these data with other spatial methods that measure the proteome [91,92], metabolome [91], or lipidome [93,94]. Addressing these questions will be essential for translating our understanding of adipocyte heterogeneity into mechanistic insights and therapeutic strategies for treatment of metabolic disease.

## Perspectives

- Adipose tissue plays an essential role in human health by regulating metabolism and energy availability, providing structural cushioning, generating heat, and communicating with other organs. Heterogeneity between adipocyte subpopulations allows adipose tissue to perform these varied functions, and some adipocyte populations may have outsized effects in metabolic disease progression.
- Recent advances in methods like snRNA-seq and spatial transcriptomics have revealed multiple populations of adipocytes with unique gene expression profiles suggestive of diverse functional roles. These subpopulations are also dynamic, and their prevalence is affected by metabolic health status (obesity, diabetes) and environmental factors (temperature, diet, etc.).
- Future work in this area is needed to understand (1) whether various adipocyte populations reflect unique cell types or cell states; (2) how each population contributes to overall adipose tissue function; and (3) how various populations drive and/or respond to metabolic disease.

## Supplementary Material

Supplementary Tables S1-S6

## Open Access

Open access for this article was enabled by the participation of University of Chicago in an all-inclusive Read & Publish agreement with Portland Press and the Biochemical Society under an agreement with Big Ten Alliance.

## Abbreviations

ASPC: adipose stromal and progenitor cell
BAT: brown adipose tissue
SAT: subcutaneous adipose tissue
scRNA-seq: single-cell RNA sequencing
snRNA-seq: single-nuclear RNA sequencing
UCP1: uncoupling protein 1
VAT: visceral adipose tissue
WAT: white adipose tissue
