# Supplementary material for: Understanding adipocyte heterogeneity across species, depot, and disease
Source: Biochem Soc Trans. 2026 Jul 24;54(8):959–72. doi: 10.1042/BST20250327 (PMC13402727; doi:10.1042/BST20250327)
Supplement: Supplementary Tables S1-S6 [file BST-2025-0327C_supp.zip › Supplemental Table-Caption.docx]

**Supplemental Table 1.** Predicted function, function-associated marker genes, depot-specificity, and associations with metabolic health among adipocyte subpopulations identified in human adipose tissue. In the depot column, “ID in” refers to subpopulations that were identified in datasets containing only one depot, and the population’s presence or absence in other depots remains unknown.

**Supplemental Table 2.** Predicted function, function-associated marker genes, depot-specificity, and associations with metabolic health among adipocyte subpopulations identified in mouse white adipose tissue. In the depot column, “ID in” refers to subpopulations that were identified in datasets containing only one depot, and the population’s presence or absence in other depots remains unknown.

**Supplemental Table 3.** Predicted function, function-associated marker genes, depot-specificity, and associations with metabolic health among adipocyte subpopulations identified in mouse brown adipose tissue. In the depot column, “ID in” refers to subpopulations that were identified in datasets containing only one depot, and the population’s presence or absence in other depots remains unknown.

**Supplemental Table 4.** A summary of published studies exploring heterogeneity in human adipose tissue.

**Supplemental Table 5.** A summary of published studies exploring heterogeneity in mouse white adipose tissue.

**Supplemental Table 6.** A summary of published studies exploring heterogeneity in mouse brown adipose tissue.
